# Supplementary material for: Prevalent gut phages encode modular adhesins mediating epithelial binding and endoplasmic reticulum trafficking
Source: Nat Commun. 2026 Jun 4;17:6191. doi: 10.1038/s41467-026-74031-x (PMC13370021; doi:10.1038/s41467-026-74031-x)
Supplement: Supplementary file 3 — Description of Additional Supplementary Files [file 41467_2026_74031_MOESM3_ESM.pdf]

Description of Additional Supplementary Files

**Supplementary Data 1.** MetaPhage output table containing mapped read counts.

**Supplementary Data 2.** Metadata of filtered phages from Metaphage output, and their abundance values after applying the Watermann formula. Data from Metaphage is highlighted with a green background.

**Supplementary Data 3.** Protein domains with putative adherence-related functions based on prior literature.

**Supplementary Data 4.** Protein domains with putative adherence-related functions based on prior literature.

**Supplementary Data 5.** List of the 13 Ig-like-bearing adherent vOTUs containing 45 ORFs that encode 67 Ig-like regions.

**Supplementary Data 6.** Gene annotations for partial sequences of the 13 adherent vOTUs with Ig-like domain-containing proteins.

**Supplementary Data 7.** Data for the network analysis of shared protein orthogroups.

**Supplementary Data 8.** CRISPR spacer matches linking vOTUs to predicted bacterial hosts

**Supplementary Data 9.** Properties of the Ig-like domain-containing proteins integrated into the K1F genome

**Supplementary Data 10.** Killing curve experiment that compares the killing efficiency of wild-type and engineered K1F phages on E. coli EV36 strain

**Supplementary Data 11.** Phage uptake experiments

**Supplementary Data 12.** Monitoring phage shedding in mouse feces

**Supplementary Data 13.** Broad Human Virome dataset, main table

**Supplementary Data 14.** 79-Individual Human Virome dataset, main table

**Supplementary Data 15.** ORF domain check

**Supplementary Data 16.** Similarity matrix showing the similarity ratio of two ORFs

**Supplementary Data 17.** Enrichment results of Ig-like domains among vOTUs from the Broad Human Virome dataset

**Supplementary Data 18.** Enrichment results of Ig-like domains among vOTUs from the 79-Individual Human Virome dataset

**Supplementary Data 19.** Enrichment results of Ig-like domains among vOTUs from the Broad Human Virome dataset filtered for vOTUs with hosts belonging to the Bacteroidota phylum

**Supplementary Data 20.** Alignment of the 50 vOTUs (queries) to representative members of the proposed Flandersviridae family (subjects)

**Supplementary Data 21.** Enrichment results of Ig-like domains among vOTUs from the Broad Human Virome dataset, associated with different diseases

**Supplementary Data 22.** The effect of different cell surface treatments on the uptake of phages by HT29-MTX epithelial cells

**Supplementary Data 23.** Colocalization experiments

**Supplementary Data 24.** Primers used for the construction of the engineered K1F phages

**Supplementary Data 25.** Results of the statistical tests
