# Supplementary material for: Prevalent gut phages encode modular adhesins mediating epithelial binding and endoplasmic reticulum trafficking
Source: Nat Commun. 2026 Jun 4;17:6191. doi: 10.1038/s41467-026-74031-x (PMC13370021; doi:10.1038/s41467-026-74031-x)
Supplement: Supplementary file 5 — Transparent Peer Review file [file 41467_2026_74031_MOESM5_ESM.pdf]

nature portfolio

Peer Review File

Prevalent Gut Phages Encode Modular Adhesins Mediating Epithelial Binding and Endoplasmic Reticulum Trafficking

Corresponding Author: Dr Gábor Apjok

This file contains all reviewer reports in order by version, followed by all author rebuttals in order by version.

Version 0:

Reviewer comments:

Reviewer #1

(Remarks to the Author)
This is a compelling and well-executed study that addresses an important and underexplored aspect of phage biology—direct interactions between bacteriophages and mammalian epithelial cells. The manuscript integrates large-scale virome analysis with functional validation in a thoughtful and effective manner, moving from computational identification of candidate adhesins to experimental demonstration of epithelial association, uptake, and altered retention in vivo.

A particular strength is the use of engineered phage scaffolds to establish causality. The demonstration that transfer of Ig-like domain-containing proteins is sufficient to confer epithelial interaction is convincing and represents a clear conceptual advance. The combination of bioinformatic analysis, in vitro assays across multiple epithelial models, and in vivo experiments further enhances the scope and impact of the work.

Overall, this study provides a meaningful contribution to our understanding of phage–host interactions and will be of broad interest to the microbiome, virology, and host–microbe interaction communities. I am supportive of publication following revision.
Major comments

1. Interpretation of in vivo retention
The mouse experiments demonstrate altered gastrointestinal retention of engineered phages, which is consistent with increased association with mucus or epithelial surfaces. However, reduced fecal recovery is not, on its own, a direct measure of epithelial adhesion or internalization.

I recommend tempering the language in this section to clarify that these data are consistent with, but do not directly establish, epithelial interaction in vivo.

If feasible, the authors could further strengthen this aspect by imaging intestinal sections following phage administration to assess localization relative to the mucus layer or epithelial surface. Even qualitative localization would provide a useful bridge between the fecal recovery data and the proposed interpretation. However, I do not view this as essential for publication provided the claims are appropriately moderated.
2. Mechanism of cellular uptake
The data clearly demonstrate enhanced uptake of adhesin-displaying phages across multiple epithelial cell types. However, the current evidence does not clearly distinguish between receptor-mediated endocytosis and other uptake mechanisms.

I do not think identification of a specific receptor is necessary for this study. However, the current phrasing (e.g., "receptor-mediated internalization") is somewhat stronger than what is directly supported by the data. I recommend modestly softening this language (e.g., "consistent with host-factor-dependent or receptor-associated uptake").

If readily feasible, the authors could optionally include simple supporting experiments (e.g., temperature dependence or competitive inhibition with soluble glycans) to further support this interpretation, though I do not consider this essential.
3. Scope and generality of claims
The bioinformatic analysis is a strong component of the manuscript and supports the conclusion that Ig-like domain-containing proteins are enriched among abundant and prevalent gut phages. However, some statements regarding the

prevalence or generality of phage–epithelial interactions could be slightly moderated to reflect:
• the relatively small number of experimentally validated adherent vOTUs
• potential biases in the screening pipeline

Framing these interactions as widespread and likely important, rather than broadly universal, would better align with the presented data.

Reviewer #2

(Remarks to the Author)
This study is valuable because it explores the function of Ig-like domains located on phage structural proteins in the affinity of phages for epithelial cells. The initial screen to select fecal phages that display some affinity on human cells is followed by virome sequencing and molecular cloning to assess the involvement of identified domains in the retention of phages in the gut using a murine model. Finally, authors extend their investigations to large virome dataset and move one step further by hypothesizing and testing that phage affinity increases the likelihood to interact and penetrate within epithelial cells. Conclusions are supported by the data.
My comments will start by reminding authors that inserting line numbers make the reviewing more rapid and accurate.

In the abstract, the term myophages is unusual. Myovirus should be used as in the rest of the paper.
In the last sentence, I would remove "in the human virome" as it does not make sense that phage-human interactions take place in the DNA sequences of viruses (definition of virome).

In the second paragraph of the introduction, the sentence including "necessitates a systematic reassessment" rings as the main justification of the study, while in the previous sentence it is already acknowledge that Ig-like domains are widespread in phage genomes. Please revise towards a functional assessment as the next sentence or even remove ?
The last sentence of this paragraph should be stopped at human host. The hypothetical link with putative therapeutic application should be kept for discussion.
In the last sentence of the third paragraph of introduction, I understand the term "targeted delivery" but it may be too strong as it suggests that phages could reach some specific location, while mucosal structures are all over the place in the gut.

In figure 1B, No Ig-like domain = 172, while in the vOTUs comparison panel N=170.
The police font at the bottom of the panel C is really too small to be informative !

In the section about identification of Ig-like domains in vOTUs authors indicate that 79% were located proximal to structural proteins, while in the methods they mention that the extended their search up to 5 ORFs in the vicinity of major or minor capsid protein. Please clarify whether such domain can be found in putatively any ORFs since these motifs seems to be present in various proteins and not exclusively structural proteins.

In figure S1, it is unclear how the morphology was assigned to the vOTUs and unless I missed it in the methods?

In the second section of Results (Displaying…) how would you know that K1F is "non-adherent". Missing reference ?

About making K1F variants, it would be easier for the readers to insert a sentence explaining the particularity of K1F gene organization with gp10 and gp10b before to mention "create an occasional translational fusion with gp29" (why gp29??). This sentence is obscure without context.

Why not cloning the vOTU_153 that seems to be so peculiar ?

In Fig S3, it is unclear why T4 titer is indicated in this figure?

I do not understand why the authors performed assays with the A549 pulmonary cells. Given the statistical difference between HT29 and Caco2 shown in panels C and D, it appears unnecessary to test A549. If this cell line was used as a kind of control, it is blurring the message about intestinal phage

In Fig 2A: add in the legend and methods the phage:cell ratio. It is critical to indicate the amount of each partner that you put in contact for these assays.
In Fig. 2C/D, were AUC calculated from the 18h time point or an earlier time point ?

In the legend for panel 2E, add "see table S12" right after the exclusion of 2 animals and in the Table S12, indicate that the red cells correspond to excluded animals likely because the gavage was not as successful compared to other animals from the same group

The term "discrepancy" is, to my point of view, not appropriate. Even if K1F::hoc does not behave like T4 (and hypothesis are correct), this phage still displays higher affinity than WT K1F. What remains surprising to me is the suitability of K1F scaffold to assess the function of Ig-like domains. This comes back to my initial remark: was K1F chosen on previous evidence that this phage does not have any affinity to cells ? It is in fact only in the next section with Ref 45 that we learn that K1F was already assessed in mice… but knowing well this study I double check and phage K1F is not used since in this paper only T4 and T7 were assessed. In fact in Fig6, there is evidence that T7 (the closer morphotype to K1F) lags in the gut of germ-

free mice for at least a week ! Perhaps there is a mistake in the reference ? Otherwise, I can suggest authors to look at this paper (PMID: 22118225) in which phage CLB_P1 is reported to be close to K1F, and in this review (PMID: 23739386) there are original data about this phage retention in mice).

Could authors explain to the readers what means 90% completeness when mentioning “high quality phage genomes”. My understanding is that these phage genomes are sufficiently similar to complete phage genomes, but there are not enough reads allowing the genome to be completed ?

In Fig3, there is an inversion of the letter B and C for the corresponding panels according to the legend.

The sentenced placed ahead of the Fig3D is difficult to understand with a succession of numbers that do not appear in the figure. You lost me !

I t would be nice to highlight in Table S9, in both AA and nucleotide sequences the mutations that differentiate ::L and ::7649

In the section “Minimal Ig-like…” can the authors discuss why T4 is not affected by any cell surface disruption, while K1F::Hoc is strongly affected ?

Overall, it is a bit annoying that different assays are performed with different cell types, preventing the possible association of observed phenotypes. For instance, ::7649 is affected in T29 cell surface disruption assay (Fig4) and is the most internalized phage on A549 internalization assays (Fig5). This resonates with the above question of why A549 cells were used in this study that is essentially focused on intestinal phages.

In the Discussion: I do not know what “ecologically consequential feature” really means…
You still use “uncharacterized Myophage” but I thought that you showed that at least some of them were close to Flandersviridae ? Is uncharacterized still accurate? This points to the question of which criteria was used to assigned these vOTU to Myovirus morphotype ?

The term “sorting decision” sounds unusual to me but could be more common in cell biology ?
The next sentence should be more ponderate: “far more complex” seems inappropriate as we knew so little so far that complexity was difficult to assess.

The sentence including “to prevent unintended interaction” is in opposition to the previous sentences proposing to exploit phage adhesion for targeted therapeutics. Either you choose a camp, or you better explain why it would be beneficial in one hand and not in the other.

What is “workings of the immune systems” ?

Another limitation is the huge gap between a very large amount of a single phage type put in contact with cells for the in vitro assays, vs, a large amount of highly variable phage particles in contact with cells embedded within an integrated organ.

One last question: any possibility to interrogate your data to determine whether phages enriched in Ig-like domain are more frequently virulent than temperate ?

Reviewer #3

(Remarks to the Author)
The results presented by the authors are important for understanding phage biology and phage interactions with the human organism. In particular, the study aims to identify the molecular determinants that enable specific phages to engage mammalian cell surfaces and cellular pathways, which remain largely unknown. However, we have substantial reservations regarding the quality, structure, and clarity of the manuscript in its current form. Below, we provide several representative examples of issues that, in our opinion, require correction. We hope that, after major revision, the manuscript will better reflect the scientific value of the study and ensure its reproducibility.
We hope that these comments will help the authors improve the clarity, rigor, and overall presentation of the manuscript.

A. Abstract
1. The first sentence (lines 26–27) is overly general and may be removed.
2. Line 31: the statement “increased phage retention in the mouse gut in vivo” should be interpreted more cautiously, because the authors did not include the appropriate controls in the relevant experiment.

B. Introduction
3. Line 68: the authors should clarify what they mean by “uncultivated gut phages”.

C. Results
4. Better separation and organization of the Results and Methods sections
In this section, it is unnecessary to repeat elements of the methodological description unless they are directly relevant to the discussion, are used to justify the choice and purpose of the method, or explain its possible influence on the result obtained. There is also no need to refer the reader to the Methods section (for example in line 98), provided that the methods are properly described there, because this is self-evident.

Overall, the manuscript requires better integration of the Results and Methods sections, or rather a clearer separation and organization of their respective elements in accordance with the principles of scientific writing. For example, the fragment “Intracellular fluorescent signal changes were tracked over 18 hours in three replicates (see Methods)” (lines 161–162) should be incorporated into the Methods section.
Conversely, the fragment “Seventeen vOTUs met these criteria, while the remaining 200 were categorized as non-adherent (‘non-adherent’ vOTU) (Figure 1B and Table S2). As expected, the T4 phage, included as a positive control, was classified as adherent” (lines 785–787) should be moved from Methods to Results, especially since the Results section currently contains only a very general summary of this analysis (lines 115–118): “In agreement with the bacteriophage adherence to mucus (BAM) model28,36, adherent vOTUs encoded Ig-like-domain-containing open reading frames (ORFs) disproportionally more frequently than non-adherent vOTUs ($p < 0.00001$, Fisher’s exact test, Fig. 1B, Table S2).” This also illustrates why the reader quite often has the impression that the descriptions of the authors’ results are too general in relation to the presented figures and supplementary tables.
5. The supplementary tables are not sufficiently reader-friendly
References to tables in the Excel files provided as Supplementary Material are, to put it mildly, not reader-friendly, because they often resemble semi-raw or working datasets rather than tables that clearly present results. In many cases, the reader must independently analyze the data contained in the cited tables in order to reconstruct the results to which the authors refer. We encourage the authors to provide “real” tables, if possible, directly in the Supplementary Material doc file.
For example, in lines 175–177, the statement “(iii) a Fibronectin type III Ig-like domain on the T4 phage tail fiber may further facilitate interaction with eukaryotic cell surface residues beyond the Ig-like domains of the Hoc protein (Table S3)” and the subsequent comments are not adequately supported by a clear presentation of the corresponding results.
Lines 154–1156: “Characterization of infection dynamics confirmed that the viability of K1F remained unaffected by the displayed Ig-like domains (Fig. S3, Table S9)” — what do the authors consider here as “viability”, because Fig. S3 and Table S9 do not show any data on K1F viability?
Lines 133–135: “Based on CRISPR spacer match predictions, these vOTUs infect Bacteroidota hosts, akin to crAss-like phages (Tables S1, S2 and S8).”
This statement is not sufficiently supported by the Methods section as currently written. The supplementary tables provide useful output data for host prediction, particularly Table S8, which appears to report SpacePHARER-based CRISPR spacer matches, and Table S1, which includes MetaPhage host assignments. However, these tables do not replace a methodological description of the host prediction workflow. At present, the reader can infer that CRISPR spacer matching was performed, but cannot determine how host assignments were ultimately made, which thresholds were applied, how conflicting or multiple hits were handled, or how the final conclusion that these vOTUs infect Bacteroidota hosts was derived. Therefore, the authors should add a dedicated Methods paragraph describing the CRISPR-based host prediction analysis in sufficient detail for the reader and reviewer to assess its validity. In addition, the wording should be softened from “infect” to “are predicted to infect” or “were linked to,” as CRISPR spacer matches support host prediction rather than direct demonstration of infection.
6. Subsection “Adhesins increase retention in the murine gastrointestinal tract”
We have reservations regarding the interpretation of the in vivo shedding experiment (Figure 2E) within the subsection “Adhesins increase retention in the murine gastrointestinal tract”. Although the results align with the authors’ expectations, bioinformatic analyses, and in vitro tests, the conclusion oversimplifies gastrointestinal phage pharmacokinetics. Equating delayed fecal recovery solely with active mucosal "retention" is rather speculative, as the lack of appropriate controls means these data cannot be regarded as fully confirming the authors’ interpretation.
The pharmacokinetics of phage elimination from the mouse gastrointestinal tract are influenced by numerous confounding factors beyond epithelial adhesion. These include:
a) Physiological variables: Host-specific factors such as gut transit time and the susceptibility of the phages to physical degradation or inactivation by gastric juices.
b) Interactions with natural flora (Phage amplification): The potential for the administered phages to interact with and replicate on the natural bacterial flora of the mice. Crucially, the manuscript does not state whether the feces of these mice were screened prior to oral gavage for native E. coli strains susceptible to the phages of interest.
c) Methodological confounders: The possible presence of endogenous phages in the mouse gut capable of lysing the specific indicator bacterial strains used to determine the fecal titers of the administered phages.
Without addressing these uncontrolled variables, the delayed fecal shedding cannot be definitively attributed to mucosal retention mediated by Ig-like domains.
7. Subsection “Phages with modular adhesins are abundant and prevalent in the healthy human virome”
A critical methodological limitation of this study is its reliance on fecal virome analysis to infer phage dynamics in IBD patients, a condition characterized by a severely compromised epithelial barrier. As established by recent research (Bollyky & Secor, Cell Host Microbe. 2019;25(2):177-179; Nguyen et al., mBio. 2017;8(6):e01874-17), bacteriophages actively translocate across the epithelium into deeper tissues—a process significantly exacerbated during mucosal inflammation. Once in the lamina propria, these translocated phages and their DNA can be internalized by immune cells, directly stimulating pro-inflammatory pathways such as TLR9-dependent IFN-gamma production, as demonstrated by Gogokhia et al. (Cell Host Microbe. 2019, 13;25(2):285-299.e8). Therefore, the observed depletion of adherent phages in the feces of IBD patients does not necessarily mean they are 'washed out' due to mucus degradation. Rather, it likely reflects their increased translocation into the ulcerated mucosa to interact directly with the underlying immune system. By ignoring the 'leaky gut' phenomenon and reducing the function of Ig-like domains strictly to mechanical adhesion in a sterile in vitro model, the authors critically overlook the broader immunomodulatory capabilities of these phages. We encourage the authors to discuss their rsults in the view of above findings.
Furthermore, the claim that these phages are signature components of a well-functioning mucosal environment based on their depletion in IBD patients is overstated. IBD inherently destroys the mucosal habitat and depletes the host bacteria, which naturally leads to phage eradication. Additionally, when utilizing the 79-Individual Gut Virome dataset to compare healthy individuals with IBD patients (Figure 3E), the authors treat IBD as a single, monolithic entity. The original dataset

explicitly provides high-resolution clinical metadata, differentiating between Crohn's disease and Ulcerative Colitis, and includes data on disease activity and medications. This correlational claim must be toned down and the available granular metadata should be rather utilized.

D. Discussion
The Discussion should be revised after revision of the Results section accordingly.

E. Supplementary Tables (S1–S23)
8. We appreciate the intention to provide as many analytical details as possible, but the material requires much better organization. In its present form, the supplementary tables resemble a better-quality draft rather than finalized supplementary material. For example, simply reorganizing the columns, starting with “Has Ig-like domain”, then “Adherence”, and only subsequently moving toward more detailed variables, would already reduce the current level of confusion. This is especially relevant in view of the enormous matrix of columns presenting “Abundance values…” with many decimal places stored in text format. Paradoxically, the values in the column “Cloned(1) or not cloned(0) gene and domains” are formatted partly as numbers and partly as text.
9. The authors have simplified their work by creating a README file in which they provide the title of each table and detailed descriptions of the columns. However, including this information directly in the top cells of each worksheet would be a substantial improvement for the reader.
10. The tables should also allow easier identification of the main analyzed parameters. For example, lines 115–118 state: “In agreement with the bacteriophage adherence to mucus (BAM) model28,36, adherent vOTUs encoded Ig-like-domain-containing open reading frames (ORFs) disproportionally more frequently than non-adherent vOTUs ($p < 0.00001$, Fisher’s exact test, Fig. 1B, Table S2).” This text does not present any numerical values describing the result. As suggested above, moving part of the relevant text from Methods to Results could help here. At present, Table S2 adds confusion because it requires the reader to perform their own analysis and transformation of the data in order to reconstruct the authors’ result.
11. Some descriptions provided for the tables contain methodological elements that should also be included in the Methods section. For example, Table S2 states: “adherent - the vOTU is considered adherent as it fulfilled both criteria: 1) was present in all four residual samples, 2) was depleted in all four effluent samples”. This definition should also be presented in Methods.
12. Table S1: morphology may still be used descriptively, but not as a formal systematic family name in the “Family” column.
13. The description of Table S13 is located in the README worksheet in a different Excel file.
14. The spreadsheet presenting Table S14 is completely empty.
15. Tables S7 and S8 have no titles.
16. Table S20: the names of the listed diseases require correction. For example, “parkinsons” is colloquial rather than a proper disease name; “artherosclerosis” should be corrected to “atherosclerosis”; “cirrhosis” should be specified more precisely, e.g. “liver cirrhosis”; “carcinoma” is too broad; “t1d” and “t2d” should both be expanded; and “spondylitis” is too general, because it refers to a group of diseases. If possible, it would be better to refer to a specific disease entity, e.g. ankylosing spondylitis.

F. Figures
The presentation of the visual data requires substantial formatting to meet accessibility and legibility standards. Please address the following issues:
17. Figure 1: The figure is currently unreadable due to overcrowding. Please increase the resolution and consider splitting it into two figures: one containing Panel A with Panel B below it (arranged horizontally), and a separate figure for Panel C at a legible size. Additionally, in Panel 1B, clarify whether "n=170" refers to the “No Ig-like domain” group, the “Detected Ig-like domain” group, or both. A pie chart is recommended here for clarity.
18. Figure 2: To reduce visual clutter, please divide this into two separate figures presenting the in vitro and in vivo results independently.
19. Figures 2, 3, and 4: These panels contain a massive amount of information. Please enlarge the text elements across all of them to ensure legibility.
20. Figure 3: The heatmap (3A) and network graph (3C) are visually dense. Please ensure high-resolution graphics, or provide a simplified schematic in the main text and move the full network to the supplementary material.
21. Figure S3B: Clarify the metric - does "log10 PFU/mL" express the AUC or the phage titer at the end of the observation period?

G. References
The reference list is structured incorrectly. While I would leave it to the Editor to decide whether the references may be accepted as two separate modules, one under the main text and another under the Methods section, restarting the numbering of the second list from 1 is not acceptable, because it confuses the reader.
22. For example, it is unclear which citation no. 7 is meant in relation to “established methodology” in line 97.

H. Methods
There are significant gaps in the description of the methodology. In several places, its current structure is highly chaotic, fragmented, and lacks critical technical details required for reproducibility.
23. Line 670: please clarify the exact pooling methodology.
24. There is no information on the origin of the E. coli EV36 strain, the E. coli BW25113 strain, or the T4 phage.
25. Lines 655–656: “Dulbecco′s Modified Eagle′s Medium supplemented with low glucose, sodium pyruvate, and GlutaMAX” would be more adequate here. This is crucial information for determining the cell culture conditions, because CACO-2 cells are, for example, usually cultivated in high-glucose DMEM, however we agree that the use of low-glucose DMEM may better model physiological conditions in the intestine.

26. There is no information on the working phage titer applied in the experiments shown in Fig. 1B. Are the phage titers used in the other experiments indicated as working titers?
27. With respect to the use of AUC in the experiments described in Fig. 1C and Fig. 4: the use of AUC does not necessarily distort the results (although nonlinear modeling would likely be preferable), provided that the discussion clearly states that the smaller AUC in this case results from slower kinetics rather than from a lower final level of binding.
28. Lines 683–688: the fragment concerning ethical issues should be separated into an independent subsection, ideally combined with the description of the study participants, including in particular the currently missing definition of “dysbiotic individuals” (on what basis they were classified as dysbiotic) and an explanation of how the remaining participants were determined to be healthy. Additionally, a new supplementary table detailing basic demographic and clinical metadata for those cohorts would be welcome.
29. Lines 785–787 – see our comments concerning the Results.
30. The sentence in lines 791–793 would be better incorporated into the Results section.
31. Subparagraph “Live cell confocal microscopy” introduce “microscopy chaos”: the description of the imaging platforms is scattered and highly confusing. It is unclear whether the colocalization data were acquired using the Operetta CLS system or the Leica Stellaris 5. The authors should consolidate these sections and explicitly link the specific microscope to the corresponding experiment and analysis.
32. Incomplete machine learning pipeline (line 950): the statement “employed supervised machine learning” is too vague. The authors must specify the exact algorithm used, the size of the training and validation datasets (e.g. number of annotated cells), and objective performance metrics (e.g. overall accuracy) on the test set. If needed the authors may deposit any step-by-step protocols used in their study in Protocol Exchange as described in Guide to authors.
33. Missing colocalization software and thresholding (lines 1010–1011): please specify which software or plugin was used to calculate the Manders' overlap coefficients. Furthermore, please state the exact “background thresholding” method used to ensure that the analysis was not subject to manual bias.
34. Lines 1034–1035: please explain how fecal samples collected from mice were diluted in PBS, including the dilution ratio.
35. Line 1039: please check whether PFU is appropriate here.
36. Line 1044: why was the T4 phage titer determined on E. coli strain BW25113 here, but not on E. coli strain EV36 as described in line 903? This could influence the comparability of the results obtained for different phages.
37. Are there any differences between “Operetta CLS high content analysis system (PerkinElmer)” and “Operetta high content analysis system”? If not, the authors may simply use “Operetta CLS system” after the first full description of this instrument in the Methods section.
38. Line 997: 10^8 PFU is an absolute number of particles, not a concentration. It should therefore be expressed, for example, as 10^8 PFU/mL. It would also be worthwhile to provide the multiplicity of infection in relation to the number of cells, or at least the cell density at the time of incubation with phages.
39. The applied statistical methods are described alongside the relevant techniques and tests, and these descriptions should ideally be supplemented with any corrections used. However, as a general rule, it would also be appropriate to include a summarizing subsection on statistical analysis stating which software was used and formally defining the significance threshold considered statistically meaningful.

Reviewer #4

(Remarks to the Author)
I co-reviewed this manuscript with one of the reviewers who provided the listed reports. This is part of the Nature Communications initiative to facilitate training in peer review and to provide appropriate recognition for Early Career Researchers who co-review manuscripts.

Open Access This Peer Review File is licensed under a Creative Commons Attribution 4.0 International License, which permits use, sharing, adaptation, distribution and reproduction in any medium or format, as long as you give appropriate credit to the original author(s) and the source, provide a link to the Creative Commons license, and indicate if changes were made.
In cases where reviewers are anonymous, credit should be given to 'Anonymous Referee' and the source.
The images or other third party material in this Peer Review File are included in the article's Creative Commons license, unless indicated otherwise in a credit line to the material. If material is not included in the article's Creative Commons license and your intended use is not permitted by statutory regulation or exceeds the permitted use, you will need to obtain permission directly from the copyright holder.
To view a copy of this license, visit https://creativecommons.org/licenses/by/4.0/

Reviewer #1 (Remarks to the Author):

This is a compelling and well-executed study that addresses an important and underexplored aspect of phage biology—direct interactions between bacteriophages and mammalian epithelial cells. The manuscript integrates large-scale virome analysis with functional validation in a thoughtful and effective manner, moving from computational identification of candidate adhesins to experimental demonstration of epithelial association, uptake, and altered retention in vivo.

A particular strength is the use of engineered phage scaffolds to establish causality. The demonstration that transfer of Ig-like domain-containing proteins is sufficient to confer epithelial interaction is convincing and represents a clear conceptual advance. The combination of bioinformatic analysis, in vitro assays across multiple epithelial models, and in vivo experiments further enhances the scope and impact of the work.

Overall, this study provides a meaningful contribution to our understanding of phage–host interactions and will be of broad interest to the microbiome, virology, and host–microbe interaction communities. I am supportive of publication following revision.
Major comments

1. Interpretation of in vivo retention
The mouse experiments demonstrate altered gastrointestinal retention of engineered phages, which is consistent with increased association with mucus or epithelial surfaces. However, reduced fecal recovery is not, on its own, a direct measure of epithelial adhesion or internalization.

I recommend tempering the language in this section to clarify that these data are consistent with, but do not directly establish, epithelial interaction in vivo.

If feasible, the authors could further strengthen this aspect by imaging intestinal sections following phage administration to assess localization relative to the mucus layer or epithelial surface. Even qualitative localization would provide a useful bridge between the fecal recovery data and the proposed interpretation. However, I do not view this as essential for publication provided the claims are appropriately moderated.

We thank the Reviewer for this important comment and agree. In the revised manuscript, we have moderated our interpretation of the murine retention data in the corresponding sections in line with the suggestion (line 208 - "*This observation is consistent with sequestration of the recombinant phages within the mucin layer and/or altered association with epithelial surfaces, although direct epithelial localization or internalization in vivo remains to be demonstrated.*"). We also removed wording that could be interpreted as implying direct in vivo epithelial interaction based on fecal shedding data alone. In accordance with the Editor's guidance, we

did not add further experiments at this stage, but we now note that direct localization of phages within intestinal sections would be an important future direction.

2. Mechanism of cellular uptake

The data clearly demonstrate enhanced uptake of adhesin-displaying phages across multiple epithelial cell types. However, the current evidence does not clearly distinguish between receptor-mediated endocytosis and other uptake mechanisms.

I do not think identification of a specific receptor is necessary for this study. However, the current phrasing (e.g., "receptor-mediated internalization") is somewhat stronger than what is directly supported by the data. I recommend modestly softening this language (e.g., "consistent with host-factor-dependent or receptor-associated uptake").

If readily feasible, the authors could optionally include simple supporting experiments (e.g., temperature dependence or competitive inhibition with soluble glycans) to further support this interpretation, though I do not consider this essential.

We thank the Reviewer and agree that our data do not resolve the precise uptake mechanism or establish receptor-mediated internalization. In the revised manuscript, we have softened the relevant wording throughout (from line 325 -"*In summary, engineered phages partially avoid lysosomal pathways and accumulate in the ER, suggesting involvement of retrograde transport, although the underlying mechanisms remain unclear.*"). In particular, we replaced stronger formulations implying receptor-mediated uptake with more cautious language indicating that the data are consistent with host-factor-dependent and/or receptor-associated uptake, while acknowledging that multiple endocytic routes remain possible. We also revised the Discussion to state more clearly that the exact entry mechanism remains unresolved and should be addressed in future work.

3. Scope and generality of claims

The bioinformatic analysis is a strong component of the manuscript and supports the conclusion that Ig-like domain-containing proteins are enriched among abundant and prevalent gut phages. However, some statements regarding the prevalence or generality of phage–epithelial interactions could be slightly moderated to reflect:

• the relatively small number of experimentally validated adherent vOTUs

• potential biases in the screening pipeline

Framing these interactions as widespread and likely important, rather than broadly universal, would better align with the presented data.

We thank the Reviewer and agree. In the revised manuscript, we have moderated statements regarding the breadth and generality of phage-epithelial interactions. We now frame these interactions as likely common and biologically important among abundant gut phages, rather

than implying a broadly universal phenomenon (such as at line 351 - "*Together, these findings indicate that epithelial and mucus adhesion may represent a recurrent ecological strategy in the human gut virome, highlighting an underexplored aspect of the gut viral community, with potential consequences for both the phage and the human host.*"). We also make it clearer that our conclusions are based on a limited set of experimentally validated adherent vOTUs and may be influenced by the selection properties of our screening pipeline (line 378 and forth - "*These conclusions should also be interpreted in light of the limited number of experimentally validated adherent vOTUs and the specific epithelial models and assay conditions used here.*").

Reviewer #2 (Remarks to the Author):

This study is valuable because it explores the function of Ig-like domains located on phage structural proteins in the affinity of phages for epithelial cells. The initial screen to select fecal phages that display some affinity on human cells is followed by virome sequencing and molecular cloning to assess the involvement of identified domains in the retention of phages in the gut using a murine model. Finally, authors extend their investigations to large virome dataset and move one step further by hypothesizing and testing that phage affinity increases the likelihood to interact and penetrate within epithelial cells. Conclusions are supported by the data.
My comments will start by reminding authors that inserting line numbers make the reviewing more rapid and accurate.

We completely agree and remedied this oversight.

In the abstract, the term myophages is unusual. Myovirus should be used as in the rest of the paper.

All instances of "myophages" have been replaced with "myovirus" as suggested by the reviewer both in the main text and on the figures.

In the last sentence, I would remove "in the human virome" as it does not make sense that phage-human interactions take place in the DNA sequences of viruses (definition of virome).

We thank the reviewer for this comment. We rephrased the sentence to avoid this ambiguity. Although "virome" is commonly used to refer to viral communities rather than only viral DNA sequences (PMID: 33785903, PMC7122492, PMID: 33785903, PMID: 26441861) we agree that the original wording could be misread in this specific sentence. We therefore replaced it with clearer wording, while retaining "virome" elsewhere where it refers to the viral community or dataset.

In the second paragraph of the introduction, the sentence including "necessitates a systematic reassessment" rings as the main justification of the study, while in the previous

sentence it is already acknowledge that Ig-like domains are widespread in phage genomes. Please revise towards a functional assessment as the next sentence or even remove ?

Completely valid point and we removed the sentence and highlighted the next one a bit more which is the genuine justification for our study (line 60): "*Critically, however, the vast majority of these Ig-like domains and other potential adhesins remain functionally uncharacterized.* "

The last sentence of this paragraph should be stopped at human host. The hypothetical link with putative therapeutic application should be kept for discussion.
In the last sentence of the third paragraph of introduction, I understand the term "targeted delivery" but it may be too strong as it suggests that phages could reach some specific location, while mucosal structures are all over the place in the gut.

At this point it is indeed premature to introduce the concept of targeted delivery, especially without proper preamble with the section discussing mucus localizing phages. Thus, agreeing with the reviewer's point, we removed this particular part of the sentence. In line 85 we changed the targeted delivery to the more general therapeutic applications: "*These findings reveal widespread phage-human interactions with implications for human health and therapeutic applications.*"

In figure 1B, No Ig-like domain = 172, while in the vOTUs comparison panel N=170. The police font at the bottom of the panel C is really too small to be informative !

Again, we are grateful for the reviewer for noticing the error, which we now corrected. We also increased the font size at the bottom of the panel C so that it is no longer illegible.

In the section about identification of Ig-like domains in vOTUs authors indicate that 79% were located proximal to structural proteins, while in the methods they mention that the extended their search up to 5 ORFs in the vicinity of major or minor capsid protein. Please clarify whether such domain can be found in putatively any ORFs since these motifs seems to be present in various proteins and not exclusively structural proteins.

We thank the Reviewer for pointing out this ambiguity. Our intention was not to imply that Ig-like domains are restricted to structural proteins. Rather, because we were specifically interested in candidate surface-exposed adhesins, we prioritized Ig-like-domain-containing ORFs located within a defined genomic neighborhood of major or minor capsid genes. We have revised the Methods and Results (line 122: "*Interestingly, the thirteen Ig-like-bearing adherent vOTUs contained multiple such domains, totaling 45 ORFs encoding 67 Ig-like regions (Table S5). Of these ORFs, 79% were located in close proximity (< 5 ORFs) to ORFs encoding structural proteins (Methods), consistent with possible association with structural elements (Fig. 1C, Table S5)"*) to clarify that "proximal to structural proteins" refers to genomic proximity within this predefined window, and not necessarily direct annotation of the Ig-like-domain-containing ORFs themselves as structural proteins.

In figure S1, it is unclear how the morphology was assigned to the vOTUs and unless I missed it in the methods?

We thank the Reviewer for pointing this out. We agree that this was not sufficiently explained. In the revised manuscript, we now clarify that the morphology labels shown in Fig. S1 were inferred from the MetaPhage output. The now-revised Methods and S1 figure legend also point to Table S1 for this information (line 604 -"*Morphotype labels shown in Fig. S1 were inferred from the MetaPhage output based on reference-based clustering and associated reference annotations, and therefore represent predicted morphotype assignments rather than morphology determined experimentally.*")

In the second section of Results (Displaying…) how would you know that K1F is "non-adherent". Missing reference ?

We thank the Reviewer for this comment. Before initiating the display experiments, we tested wild-type K1F on epithelial cells and observed no detectable internalization under our assay conditions. Because wild-type K1F was subsequently used throughout the study as the baseline/negative reference, and this behavior was consistently reproduced in the uptake experiments presented in Fig. 2 and Table S11, we did not include the initial preliminary experiment separately. We have revised the manuscript to clarify that the term 'non-adherent' refers specifically to the low-interaction/minimal-internalization behavior of K1F under the epithelial assay conditions used here, line 620 - "*K1F was included as a low-interaction baseline control based on preliminary testing under the same epithelial uptake assay conditions and its consistent behavior across the experiments presented here.*"

About making K1F variants, it would be easier for the readers to insert a sentence explaining the particularity of K1F gene organization with gp10 and gp10b before to mention "create an occasional translational fusion with gp29" (why gp29??). This sentence is obscure without context.

We agree that the original wording did not provide enough context for readers unfamiliar with the K1F display system. In the revised manuscript, we added sentences explaining that the inserts were introduced at the 3′ end of the minor capsid gene, which is produced through a programmed translational frameshift from the major capsid gene (Line 150 -"*Specifically, in K1F, gene10 encodes the major capsid protein gp29, while a programmed -1 translational frameshift in the 3′ region generates an extended product, referred to as gene10b, which functions as the minor capsid protein. By placing the insert at this locus, the displayed proteins are fused only to the frameshift-derived minor capsid product and are therefore incorporated into only a subset of capsid subunits, minimizing disruption to phage assembly and function*"). This design results in fusion of the displayed adhesins to only a subset of capsid subunits, thereby enabling surface display while minimizing disruption to phage assembly. We also revised the wording around the fusion partner to make this logic clearer.

Why not cloning the vOTU_153 that seems to be so peculiar ?

We agree that vOTU_153 is a peculiar and potentially informative case, particularly because it carries a single Big2-type Ig-like domain rather than the more recurrent modular architectures observed in the other adherent vOTUs. During candidate selection, we recovered the corresponding determinant by PCR from the fecal viral filtrate, but we were unable to establish a viable engineered K1F variant displaying this insert. While the precise reason remains unresolved, this may reflect incompatibility of the determinant with the K1F display context. We therefore proceeded with the remaining candidates that could be experimentally implemented.

In Fig S3, it is unclear why T4 titer is indicated in this figure?

We thank the Reviewer for pointing this out and we have therefore removed the T4 titer from the revised figure.

I do not understand why the authors performed assays with the A549 pulmonary cells. Given the statistical difference between HT29 and Caco2 shown in panels C and D, it appears unnecessary to test A549. If this cell line was used as a kind of control, it is blurring the message about intestinal phage

We agree that the rationale for including A549 cells required clearer explanation. Our aim was not to investigate exclusively gut-restricted phages, but to identify phages and genetic determinants capable of mediating epithelial interaction, using pooled fecal viral filtrates as a diverse source of candidate phages. A549 cells were included as a distinct epithelial model with high uptake capacity, allowing us to test whether adhesin-mediated uptake was reproducible beyond intestinal epithelial lines and not restricted to a single gut-derived model. We also acknowledge that using different epithelial cell types across assays limits direct phenotype-to-phenotype comparisons, particularly for comparisons such as K1F::7649 in HT29-MTX perturbation assays versus A549 colocalization assays. We therefore interpret such cross-assay comparisons cautiously.

In Fig 2A: add in the legend and methods the phage:cell ratio. It is critical to indicate the amount of each partner that you put in contact for these assays.

For clarity, we now specify the phage:cell ratio in both the Fig. 2A legend and the Methods. In these assays, approximately 10^8 phages were applied to ~3 * 10^4 cells, corresponding to an average phage:cell ratio of ~10,000:1.

In Fig. 2C/D, were AUC calculated from the 18h time point or an earlier time point ?

AUC values in Fig. 2C and 2D were calculated over the full 18-hour measurement period, i.e. using the entire uptake curve from the start of the assay to the 18 h endpoint. This information is now introduced into the legend.

In the legend for panel 2E, add "see table S12" right after the exclusion of 2 animals and in the Table S12, indicate that the red cells correspond to excluded animals likely because the gavage was not as successful compared to other animals from the same group

We thank the Reviewer for this helpful suggestion. We have revised the legend for Fig. 2E to include "see Table S12" immediately after mentioning the exclusion of two animals. We have also updated Table S12 to indicate more clearly which entries correspond to the excluded animals and added an explanatory note clarifying that these exclusions likely reflect less successful gavage compared with the other animals in the same group.

The term "discrepancy" is, to my point of view, not appropriate. Even if K1F::hoc does not behave like T4 (and hypothesis are correct), this phage still displays higher affinity than WT K1F. What remains surprising to me is the suitability of K1F scaffold to assess the function of Ig-like domains. This comes back to my initial remark: was K1F chosen on previous evidence that this phage does not have any affinity to cells ? It is in fact only in the next section with Ref 45 that we learn that K1F was already assessed in mice… but knowing well this study I double check and phage K1F is not used since in this paper only T4 and T7 were assessed. In fact in Fig6, there is evidence that T7 (the closer morphotype to K1F) lags in the gut of germ-free mice for at least a week ! Perhaps there is a mistake in the reference ? Otherwise, I can suggest authors to look at this paper (PMID: 22118225) in which phage CLB_P1 is reported to be close to K1F, and in this review (PMID: 23739386) there are original data about this phage retention in mice).

We thank the Reviewer for this careful comment. We agree that the term "discrepancy" was not the best choice in this context and have replaced it with "difference." We also agree that the reference was used inappropriately in relation to wild-type K1F. As the Weiss et al. study does not directly present K1F, we revised the corresponding part accordingly so that it now refers more precisely to closely related phages, rather than implying prior direct evidence specifically for K1F.

K1F was chosen as the engineering scaffold for practical and experimental reasons: it is readily amenable to genome engineering and was available in our laboratory system. Before initiating the display experiments, we tested wild-type K1F in our epithelial uptake assay framework and found that it showed minimal internalization under the assay conditions used here. Because this low-interaction behavior was then consistently reproduced in the experiments presented throughout the study, we used K1F as the baseline scaffold/reference throughout the study. To avoid overgeneralizing this point, we reduced categorical use of "non-adherent" and clarified that this designation refers specifically to the low-interaction / minimal-internalization behavior of wild-type K1F under the epithelial assay conditions used in this study (line 144 - *"To validate the predicted function of the identified Ig-like domain-containing proteins, we*

displayed a subset of them on the surface of the Escherichia coli phage K1F scaffold, which showed minimal or undetectable internalization under the epithelial assay conditions used here and was therefore used as a non-adherent baseline.")

As a general point of comparison, Weiss et al. reported that the related podophage T7 showed rapid gut transit in germ-free mice and became undetectable in feces within approximately one day. In our study, wild-type K1F similarly showed rapid gastrointestinal transit, with fecal phage counts peaking at approximately 4 h post-administration, whereas the engineered K1F variants displayed markedly reduced fecal recovery relative to wild-type K1F.

At the same time, we agree that persistence of related podophages in the gut can depend strongly on the experimental model and host availability. We therefore do not interpret the lagging/persistence reported for T7 or other K1F-like phages in previous studies as direct evidence for epithelial adherence or internalization. Instead, our conclusion is limited to the data presented here: display of Ig-like-domain-containing adhesins on the K1F scaffold alters epithelial uptake in vitro and gastrointestinal retention dynamics in vivo relative to wild-type K1F.

Could authors explain to the readers what means 90% completeness when mentioning "high quality phage genomes". My understanding is that these phage genomes are sufficiently similar to complete phage genomes, but there are not enough reads allowing the genome to be completed ?

In the revised manuscript, we now explicitly explain that completeness was estimated using CheckV and refers to the predicted fraction of the expected viral genome represented by the assembled contig. The new text is in line 106 - "*....vOTUs were filtered for quality based on genome completeness (≥90%, that is, the predicted fraction of the expected viral genome captured by the assembled contig) and coverage (≥1, that is, the mean read coverage across all positions in the contig),....*". Accordingly, genomes with >90% completeness were treated as high-quality / near-complete, although not necessarily fully closed genomes. To be consistent, we explained coverage, too.

In Fig3, there is an inversion of the letter B and C for the corresponding panels according to the legend.

This was a legend-order issue rather than a labeling error in the figure itself. The panel letters in Fig. 3 were already correct as displayed, but the order of panels B and C in the legend was inconsistent with the figure. We have revised the legend accordingly so that it now matches the figure and the order in which the panels are discussed in the text.

The sentenced placed ahead of the Fig3D is difficult to understand with a succession of numbers that do not appear in the figure. You lost me !

To improve readability, we rewrote the sentence preceding Fig. 3D in line 258**.**

It would be nice to highlight in Table S9, in both AA and nucleotide sequences the mutations that differentiate ::L and ::7649

This comment helped us identify an error in the manuscript text. For clarity, Table S9 has been revised to highlight the sequence differences between K1F::L and K1F::7649 at both the amino acid and nucleotide levels within the corresponding sequences, and a short explanatory note has also been added directly to the table. Specifically, we now indicate the I375T substitution together with the altered C-terminal region, where K1F::L ends in VFNLG and K1F::7649 ends in YWA*. In addition, we corrected the corresponding wording in the manuscript, which had previously and incorrectly described these variants as differing by only two amino acids.

In the section "Minimal Ig-like…" can the authors discuss why T4 is not affected by any cell surface disruption, while K1F::Hoc is strongly affected ?

We have now addressed it more explicitly in the revised manuscript. We agree that the different behavior of native T4 and K1F::Hoc following cell-surface treatments likely reflects scaffold-dependent effects, rather than the activity of the Hoc alone. In the revised text (starting at line 293 - "*As detailed above, this difference likely stems from scaffold-dependent factors rather than Hoc alone: native T4 carries more Hoc molecules, presents Hoc in its native capsid context, and also includes an additional Fibronectin type III Ig-like domain that was not transferred to K1F.*"), we now point readers to the considerations already raised earlier in the Results and briefly reiterate them in this section. Specifically, native T4 carries a substantially higher number of Hoc molecules than the engineered K1F::Hoc particle, the capsid-binding region of Hoc is adapted to the native T4 capsid, and T4 may also benefit from additional surface features beyond Hoc itself, including a Fibronectin III Ig-like domain. We therefore now make it clearer that transfer of Hoc onto K1F demonstrates functional transferability, but does not necessarily recreate the full native interaction behavior of T4.

Overall, it is a bit annoying that different assays are performed with different cell types, preventing the possible association of observed phenotypes. For instance, ::7649 is affected in T29 cell surface disruption assay (Fig4) and is the most internalized phage on A549 internalization assays (Fig5). This resonates with the above question of why A549 cells were used in this study that is essentially focused on intestinal phages.

We acknowledge that the use of different cell lines in the final sections may make direct comparisons less straightforward. However, our aim was not to investigate exclusively gut-restricted phages per se, but rather to take a more exploratory approach to identify phages, and the corresponding genetic determinants, capable of interacting with epithelial cells, using pooled fecal viral filtrates as a diverse source of candidate phages. Additionally, we systematically compared epithelial adherence and uptake across all three cell lines for the full panel of engineered phages. In these experiments, the different Ig-like domains influence phage uptake in a highly consistent manner in both A549 and HT29-MTX cells, supporting the

robustness of the observed effects across epithelial models. For this reason, A549 cells were included as a distinct epithelial model with high mucus production and elevated uptake capacity, rather than as an intestinal surrogate. This decision was also driven by practical considerations: HT29-MTX cells grow considerably more slowly, and extending these experiments to this model would have required several additional months. This is particularly relevant given the extensive optimization needed to reliably visualize colocalization with multiple phages and subcellular dyes.

In the Discussion: I do not know what "ecologically consequential feature" really means…

This wording was too vague. We replaced "ecologically consequential feature" with clearer and more specific language in the revised Discussion at line 331 "*play a role in shaping the gut virome ecology*".

You still use "uncharacterized Myophage" but I thought that you showed that at least some of them were close to Flandersviridae ? Is uncharacterized still accurate? This points to the question of which criteria was used to assigned these vOTU to Myovirus morphotype ?

Fair point. We have revised the part in question to make both the taxonomic and morphotype interpretation more precise. The vOTUs in this cluster were not confidently assigned to a family by vConTACT3, which is why we originally described them as uncharacterized. However, we have performed an additional nucleotide similarity analysis against phages categorized as belonging to the recently proposed Flandersviridae family. This analysis showed that most of the 50 vOTUs share >90% nucleotide similarity with members of this proposed group, while the remaining vOTUs showed slightly lower but still substantial similarity. We therefore revised the manuscript to describe this cluster as closely related to the proposed Flandersviridae group, rather than as formally assigned members of that family. Additionally, we have added a new supplementary table (Table S20) reporting the nucleotide similarity results for these 50 vOTUs. Regarding the myovirus wording, we also clarified in the corresponding Methods section that the morphotype assignment was based on MetaPhage predictions and sequence similarity with known morphotype features. We recognize that Myo-, Sipho- and Podoviridae are no longer valid formal taxonomic families, but the corresponding morphotypes remain valid. Lastly, we cite this paper at the end of this sentence: (https://link.springer.com/article/10.1186/s40168-021-01017-w).

The term "sorting decision" sounds unusual to me but could be more common in cell biology ?
The next sentence should be more ponderate: "far more complex" seems inappropriate as we knew so little so far that complexity was difficult to assess.

The Reviewer is right that this wording was not ideal. We replaced "sorting decision" with a more standard expression ("*intracellular routes*" in line 353) and moderated the following sentence (line351 - *"Together, these findings indicate that epithelial and mucus adhesion may represent a recurrent ecological strategy in the human gut virome, highlighting an underexplored aspect of the gut viral community, with potential consequences for both the phage and the human host."*) to avoid overstating the level of established complexity.

The sentence including "to prevent unintended interaction" is in opposition to the previous sentences proposing to exploit phage adhesion for targeted therapeutics. Either you choose a camp, or you better explain why it would be beneficial in one hand and not in the other.

We revised this section to distinguish between intentional engineering of phage-host interactions for delivery applications (line 370 - *"As the technology matures and safety data accumulate, engineered adhesins on phage surfaces could be leveraged to enhance tissue targeting, retention, or cellular uptake, potentially enabling targeted intracellular delivery or immune modulation").*

What is "workings of the immune systems" ?

We removed this unclear expression from the revised manuscript.

Another limitation is the huge gap between a very large amount of a single phage type put in contact with cells for the in vitro assays, vs, a large amount of highly variable phage particles in contact with cells embedded within an integrated organ.

We added this point to the limitations section (line 380 - *"Another limitation is the difference between the in vitro and in vivo settings: (i) cultured cells were exposed to a high abundance of a single phage, whereas tissues in vivo encounter a diverse phage population and (ii) we do not yet know under which physiological conditions epithelium-associated phages undergo internalization in the human gut in vivo, where mucus architecture and immune activity differ substantially from in vitro systems.")*. The revised text now states that cultured cells were exposed to a high abundance of a single phage type, whereas tissues in vivo encounter diverse phage populations within an integrated organ environment.

One last question: any possibility to interrogate your data to determine whether phages enriched in Ig-like domain are more frequently virulent than temperate ?

We addressed this question by testing whether predicted lifestyle was associated with Ig-like domain content in the Broad Human Virome dataset. This analysis did not reveal a statistically significant association between Ig-like domain presence and predicted lytic or temperate lifestyle (Fisher's exact test, $p = 0.2456$). We have added the following sentence to the revised manuscript: "Using BACPHLIP-predicted lifestyles, Ig-like domain carriage was not

significantly associated with a predicted lytic or temperate lifestyle (Fisher's exact test, $p = 0.2456$; Table S14)." Supplementary Table S14 has also been updated to include the BACPHLIP-predicted lifestyle calls used for this analysis. The Methods section was also updated with the sentence: "*When investigated, phage lifestyle was predicted using BACPHLIP. Phages were classified as virulent or temperate only when the corresponding predicted probability exceeded 0.8.*"

Reference: https://pmc.ncbi.nlm.nih.gov/articles/PMC8106911/
